# Supplementary material for: Mechanisms for <100> interstitial dislocation loops to diffuse in BCC iron
Source: Nat Commun. 2021 Jan 11;12:225. doi: 10.1038/s41467-020-20574-6 (PMC7801622; doi:10.1038/s41467-020-20574-6)
Supplement: Supplementary file 1 — Supplementary Information [file 41467_2020_20574_MOESM1_ESM.pdf]

Supplementary Information

Mechanisms for <100> Interstitial Dislocation Loops to Diffuse in BCC Iron

N. Gao[1,2], Z. W. Yao[3,4*], G. H Lu[5], H. Q. Deng[6] and F. Gao[7,8*]

[1]Institute of Frontier and Interdisciplinary Science and Key Laboratory of Particle Physics and Particle Irradiation (MOE), ShanDong University, QingDao, 266237, China

[2]Institute of Modern Physics, Chinese Academy of Sciences, LanZhou, 730000, China

[3]Key Laboratory of Bionic Engineering Ministry of Education, Jilin University, Changchun 130022, PR China

[4]Department of Mechanical and Materials Engineering, Queen's University, Kingston, ON K7L3N6, Canada

[5]Department of Physics, Beihang University, Beijing 100191, China

[6]School of Physics and Electronics, Hunan University, Changsha 410082, China

[7]Department of Nuclear Engineering and Radiological Sciences, University of Michigan, Ann Arbor, MI 48109, USA

[8]Department of Materials Science and Engineering, University of Michigan, Ann Arbor, MI 48109, USA

[*]To whom correspondence should be addressed:

Z.W.Y.: zhongwen.yao@gmail.com and F. G.: gaofeium@umich.edu

Supplementary Notes

Supplementary Note 1. Image force tested by method suggested by Wei Cai, Ju Li and Sidney Yip [1].

Following this method, after moving the dislocation loop an equivalent lattice site along its Burgers vector direction in the present simulation box (20 unit cells along each direction), the formation energy has been calculated according to equation (2). The results are shown in supplementary Fig.1, which includes the comparison of a <100> loop with different habit planes. All these results clearly show the change of the formation energy can be neglected. Thus, according to this theory, image force on the dislocation from the boundary condition can be neglected with the given size of the simulation box used in this work.

Furthermore, according to the equation developed by Cai et.al. for periodic image effect (eq.19) in paper [2] and energy for prismatic dislocation loop (eq. 1 and C.3) in paper [3]

$$E_{img} = \frac{1}{2}\sum_R\left[E_{dd}(R) - E_{dg}(R)\right] - \frac{1}{2}E_{dg}(R=0) \sim \frac{\mu b^2 F(\nu)}{(1-\nu)R^3} \quad (1)$$

$$E^{\infty} = 2\pi r\frac{\mu b^2}{4\pi(1-\nu)}(ln\frac{8r}{r_c} - 1) \quad (2)$$

Where r is the radius of loop, r_c is the dislocation core radius, b is the value of Burgers vector, μ is shear modulus and ν is Poisson's ratio. For bcc iron, $F(\nu)$ is around 1.0 according to the calculation in Cai's paper with ν around 0.3. R is the radius of cylinder to include the dislocation loop, which can be approximated by half of computational box length. Although this approximation would introduce some uncertainty from a cylinder to a cube box, the dependence of image energy on $1/R^3$ would be kept according to Cai's theory since the image energy can be obtained as a sum of interaction between the primary loop and its periodic images, which is a function of $1/R^3$ no matter the loop in cylinder or cube computational box. Thus, according to these two equations, given the loop with radius of 1.0 nm, box length of 5.71nm and r_c around the value of b, the ratio between the image and self energy of dislocation loop studied in this work is around 3.6×10^{-6}, which can be neglected for the present work under periodic boundary condition. In fact, the above ratio calculated by H. J. Hu et.al. for a dislocation loop in HCP lattice (Fig.8 in paper [4]) has also proved our conclusion that image force can be neglected with the size of box used in the present work.

Supplementary Note 2. Saddle point states explored by NEB.

In Fig.4 in the main text, there are saddle point states between the {100}, {130}, {120} and {110} habit planes. The configurations of these states are shown in supplementary Fig.2. It is clear that these states are also possible metastable habit planes, which may be not expressed exactly by the integers {hk0}, but are still located between different habit planes. However, the saddle point states are explored by the SAAMD and used by the NEB method to determine the energy barriers.

Supplementary Note 3. Introduction of SAAMD method and determination of related parameters

The boost potential takes the following format (eq.3), which could be obtained from the state of atom on its harmonic vibration around its stable atomic position, as we explained in paper [5].

$$V_{bias}(\{r_1, \dots \dots r_{N_{AV}}\}; t) = E_b(t)\left\{1 - \left[\frac{\xi(\{r_1, \dots \dots r_{N_{AV}}\})}{q(t)}\right]^2\right\} H[q(t) - \xi(\{r_1, \dots \dots r_{N_{AV}}\})] \quad (3)$$

where E_b and q are parameters, as a function of simulation time t. ξ is total displacement of atoms in active volume (AV) within the system. Here, AV is defined as a cylinder around the <100> loop with a radius 20% larger than that of the loop. H is the Heavyside step-function to ensure when atoms in the AV are far away from equilibrium, the boost potential becomes zero. Therefore, the boost potential developed in this work provides a way to keep the system vibrating around at the state determined by $q(t)$. The self-adaptive feature of SAAMD is that E_b and q evolve with time. The underlying physics for this self-adaptive is to make sure the system evolve from the local minimum state to higher energy state gradually. The initial values of E_b and q start at small values, initially yielding a very modest bias. In this work, the E_b starts from 1 eV and q starts from ξ_0+0.1Å, where ξ_0 is the initially total displacement of atoms due to their vibrations at a given temperature before applying the boost potential. It should be noted that the large Δq and ΔE_b would result in the significant fluctuations of the transition time from one state to another, thus leading to poor statistics. Following the transition state theory and previous AMD algorithms, V_{bias} should be limited to within small value (e.g. less than 0.5 eV for around total 1700 atoms in activation volume

in the present work, i.e. 0.00029 eV each atom) to void the significant change of energy surface but keep the high computational efficiency, according to our experiences, for rugged energy surface. Thus, Δq is generally taken around 1% of the initial total displacement ξ, that is around 3.0 Å in this work. According to equation 3, ΔE_b can be determined as around 2.5 eV to 3.0 eV. In this way, the increase of E_b can restrain the total displacement ξ close to q(t) after applying the boost potential, to ensure the system changes smoothly. The stabilities of these parameters have also been confirmed by our SAAMD method by showing the fluctuation of time as shown in supplementary Fig.3

Furthermore, after carefully following the diffusion of one <100> crowdion in a <100> loop, we can determine the maximum displacement (D_{max}) to stop the boost. As shown in supplementary Fig. 4, when a <100> loop initiates its rotation to other habit plane, it needs to move the mass center of its <100> crowdion away from the original site. If this movement is limited, the loop remains, because the <100> crowdions would move forward and backward from its original site, without leading to the diffusion of the loop. Thus, as shown in supplementary Fig. 4, for the rotation of a [100] loop from its original habit plane (100) to (-310), the mass center of one [100] dumbbell should move from R1 region (close to atom *a* and *b*) to R2 region (close to atom *4* and *5*), which requires the movement of atom *a* to *1*, *b* to *2*, *c* to *3*, *d* to *4*, *e* to *5* and *f* to *6*. Here, atoms *a* to *f* are original sites and *1* to *6* are new sites after the dumbbell movement. In order to reach such a movement, the maximum distance from atom *c* to *3* should be satisfied. This value is around 2.4 Å. Considering the effect of thermal fluctuation during the diffusion of loop, the D_{max} should be less than 2.4 Å. We then did the pre-simulation from D_{max} = 2.4 Å and decreased it gradually to test whether the system could overcome the saddle point state by SAAMD simulations. After enough pre-simulations, we determined that the value of D_{max} around 2 Å is large enough to trigger the rotation and result in the same diffusion process observed from in-situ TEM.

Supplementary Note 4. Movie-1: **Diffusion of a <100> loop from SAAMD simulations.**

The atomic diffusion process of a <100> loop in BCC iron is illustrated through the self-adaptive accelerated molecular dynamics simulations at 300 K. In this movie, the dislocation loop (the red circle) is identified by DXA method applied in Ovito software. In addition, only the high energy atoms related with the loop core are displayed. The three directions of computational box are along the [100], [010] and [001], respectively. The <100> loop diffuses through the change of its habit

plane between the {100} and the {110} planes through the immediate states with the {120} and {130} habit planes. The mixed habit plane is also observed during its diffusion. The total diffusion distance is around 4.5 nm within ~ 2.65 μs.

Supplementary Note 5. Movie-2: **Direct evidence of a <100> loop diffusion with in-situ TEM observation.**

The atomic diffusion process of a <100> loop in a single crystal iron at 773 K irradiated with 150 keV Fe^{+} ions is shown through the *in-situ* irradiation and TEM measurements. In the movie, the diffusion of a <100> loop located at the middle of the top region is clearly shown. During its diffusion, the rotation of the loop from its habit plane of the {100}, to the {130}, to the {120}, and to the {110} has been observed. It is of interest to note that the loop also combines with another <100> loop to form a larger loop, which continues to diffuse via mixed habit planes, as shown in the movie. All these results confirm the diffusion process explored by SAAMD simulations.

Supplementary Note 6. Movie-3: Diffusion of a <100> loop to free surface observed by in-situ TEM method.

The diffusion of two <100> loops to a free surface is illustrated experimentally in the movie, which provides an indirect evidence to demonstrate the diffusion of <100> loops. The two loops are located at the middle region of the movie. With increasing time, the brightness of different sections of each loop changes between dark and bright. At the end of the movie, two loops are both absorbed by the near free surface. According to the TEM diffraction contrast theory, if the habit plane of a <100> loop remains the same, the brightness of the loop should be always the same. The brightness change of the loop during its diffusion to the near free surface clearly shows the diffusion of <100> loops.

Supplementary Figures

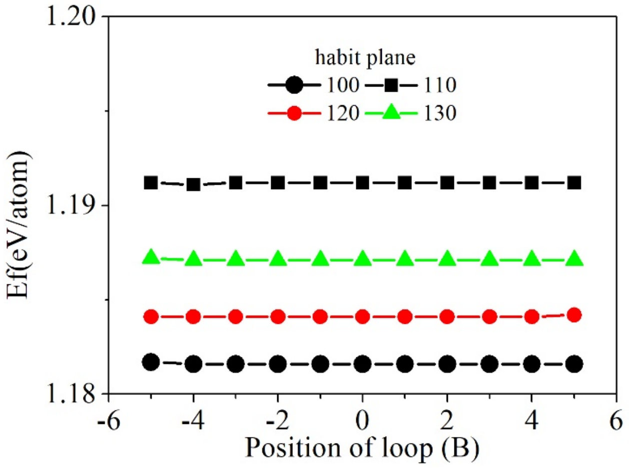

Supplementary Figure 1| Formation energy of a <100> dislocation loop with different habit plane located at different position along its Burgers vector. The unit of distance is defined as the value of Burgers vector.

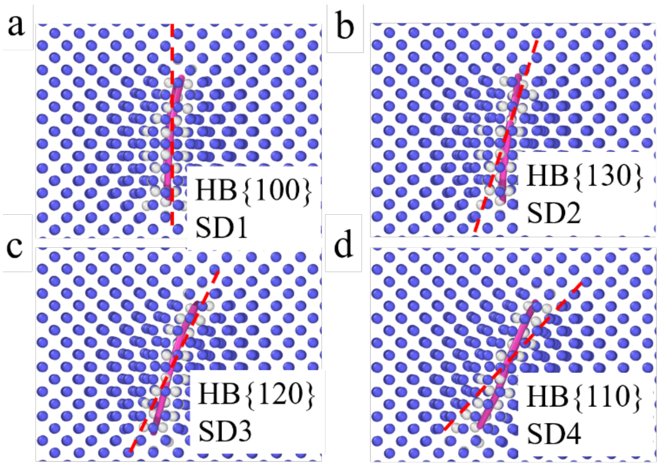

Supplementary Figure 2| Configurations of saddle point states during the rotation of the habit planes from the {100} to the {110}. For clarity, the habit planes of the {100}, {130}, {120} and {110} are represented by the red dash lines. The loop is represented by the pink curves.

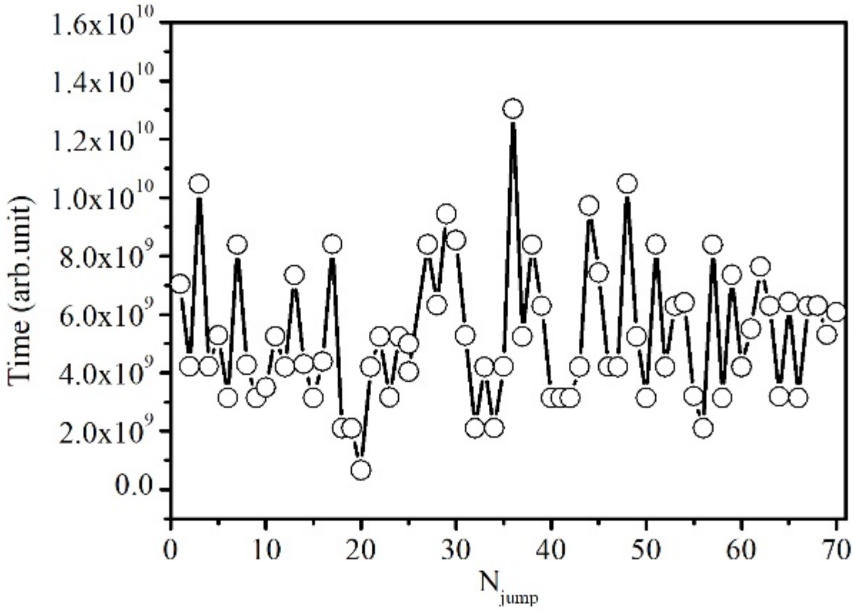

Supplementary Figure 3| Time Fluctuation of each jump for a <100> loop diffusion with Δq ~ 3.0 Å and ΔE_b ~ 2.5 eV. The stabilities of parameters used in the present simulations have been confirmed by above results.

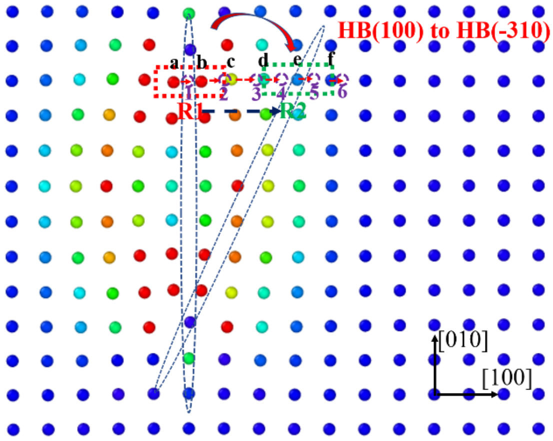

Supplementary Figure 4| Schematic to determine the theoretical maximum displacement D_{max}. The rotation of a [100] loop from its original habit plane (100) to (-310), the mass center of one [100] dumbbell should move from R1 region (close to atom *a* and *b*) to R2 region (close to atom *4* and *5*), which requires the movement of atom *a* to *1*, *b* to *2*, *c* to *3*, *d* to *4*, *e* to *5* and *f* to *6*.

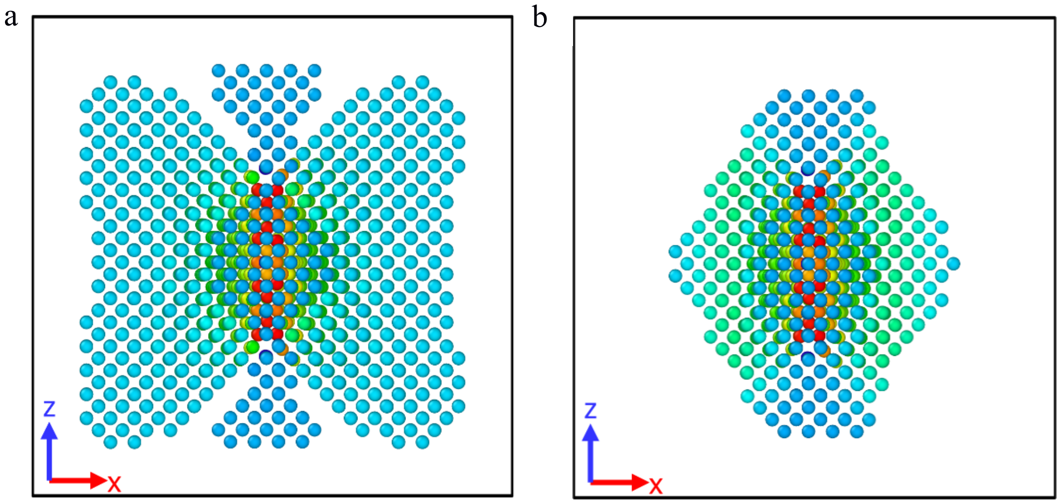

Supplementary Figure 5| Stress distribution of a <100> loop with radius of 1.0 nm. The computational box has 20 unit cells along each direction. The stress distribution along X direction is shown in (a) and along Z (or Y) direction is shown in (b). In the figure, only the atoms with stress difference higher than 0 from the perfect atom are shown. Therefore, the box size used in this work is large enough to avoid the interaction between loop and its image.

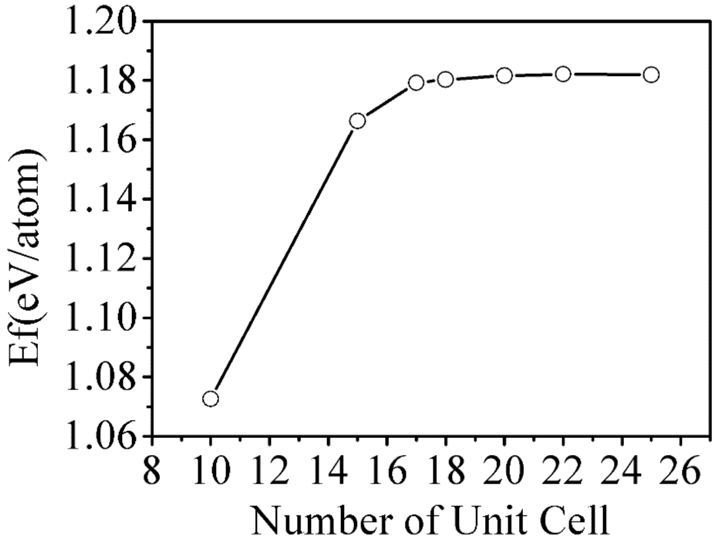

Supplementary Figure 6| Size effect on formation energy of loop as a function of unit cells of computational box. The radius of loop is 1.0 nm. It is clear the size effect on formation energy can be ignored when the number of unit cells is larger than 17~18 for a <100> loop with radius of 1.0 nm.

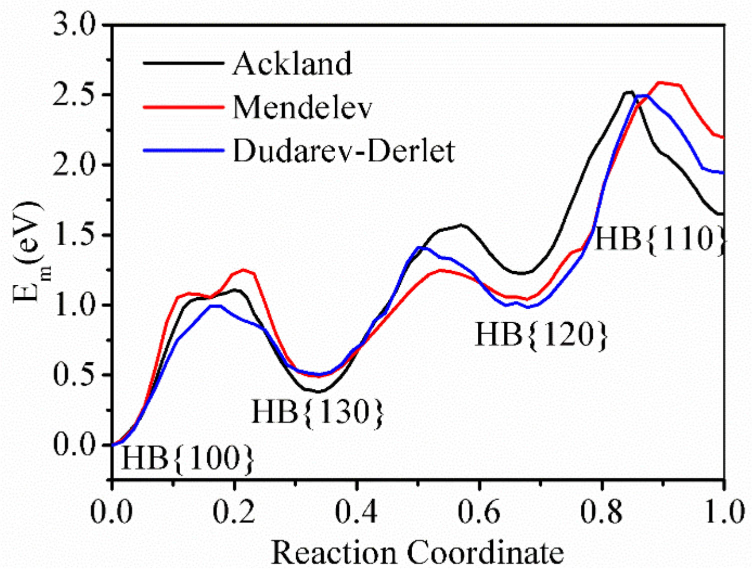

Supplementary Figure 7| Energy barrier calculated with Ackland04 [6], Mendelev [7] and Dudarev-Derlet [8] Fe potentials for a <100> loop with a diameter of around 1.6 nm. From this figure, although the exact energy barrier is different from different potentials, the general trend during the rotation is similar from these three potentials.

Supplementary References

1. Cai, W., Li, J. & Yip, S. Molecular Dynamics. In: R. J. M. Konings(ed.) Comprehensive Nuclear Materials, volume 1, pp 249-265 Amsterdam: Elsevier

2. Cai, W. Periodic image effects in dislocation modelling. *Philos. Mag.* **83**, 539-567 (2003)

3. Cai, W. & Weinberger, C. R. Energy of a Prismatic Dislocation Loop in an Elastic Cylinder. *Math. Mech. Solids.* **14**, 192-206 (2009)

4. Chu, H. J. et al. Elastic fields of dislocation loops in three-dimensional anisotropic biomaterials. J. Mech. Phys. Solids. **60**, 418-431(2012)

5. Gao, N. et al. Long-time atomistic dynamics through a new self-adaptive accelerated molecular dynamics method. *J. Phys. Condens. Matter.* **29**,145201 (2017).

6. Ackland, G. J. et al., Development of an interatomic potential for phosphorus impurities in α-iron. *J. Phys. Condens. Matter.* **16**, S2629 (2004).

7. Mendelev, M. I. et al., Development of new interatomic potentials appropriate for crystalline and liquid iron. *Philos. Mag.* **83**, 3977-3994 (2003).

8. Dudarev, S. L. & Derlet, P. M. A 'magnetic' interatomic potential for molecular dynamics simulations. *J. Phys. Condens. Matter.* **17**, 7097-7118 (2005).
