# Supplementary material for: Mechanisms for <100> interstitial dislocation loops to diffuse in BCC iron
Source: Nat Commun. 2021 Jan 11;12:225. doi: 10.1038/s41467-020-20574-6 (PMC7801622; doi:10.1038/s41467-020-20574-6)
Supplement: Supplementary file 3 — Description of Additional Supplementary Files [file 41467_2020_20574_MOESM3_ESM.pdf]

Description of Additional Supplementary Files

File Name: Supplementary Movie 1
Description: Diffusion of a <100> loop from SAAMD simulations.

File Name: Supplementary Movie 2
Description: Direct evidence of a <100> loop diffusion with in-situ TEM observation.

File Name: Supplementary Movie 3
Description: Diffusion of a <100> loop to free surface observed by in-situ TEM method.
